# A Biomimetic Electrospun Membrane Supports the Differentiation and Maturation of Kidney Epithelium from Human Stem Cells

**DOI:** 10.3390/bioengineering9050188

**Published:** 2022-04-26

**Authors:** Xingrui Mou, Jessica Shah, Rohan Bhattacharya, Titilola D. Kalejaiye, Bowen Sun, Po-Chun Hsu, Samira Musah

**Affiliations:** 1 Department of Biomedical Engineering, Pratt School of Engineering, Duke University, Durham, NC 27708, USA; xingrui.mou@duke.edu (X.M.); jessica.shah@duke.edu (J.S.); rohan.bhattacharya@duke.edu (R.B.); titilola.kalejaiye@duke.edu (T.D.K.); 2 Center for Biomolecular and Tissue Engineering, Duke University, Durham, NC 27708, USA; 3 Thomas Lord Department of Mechanical Engineering and Material Sciences, Pratt School of Engineering, Duke University, Durham, NC 27708, USA; bowensun56@163.com (B.S.); pochun.hsu@duke.edu (P.-C.H.); 4 Division of Nephrology, Department of Medicine, Duke University School of Medicine, Durham, NC 27710, USA; 5 Department of Cell Biology, Duke University, Durham, NC 27710, USA; 6 Affiliate Faculty of the Developmental and Stem Cell Biology Program, Duke Regeneration Center, Duke MEDx Initiative, Duke University, Durham, NC 27710, USA

**Keywords:** silk fibroin, electrospinning, stem cells, human induced pluripotent stem cells, podocytes, biomaterials, tissue engineering, kidney tissue engineering, in vitro models, biomimetic membranes

## Abstract

Podocytes derived from human induced pluripotent stem (hiPS) cells are enabling studies of kidney development and disease. However, many of these studies are carried out in traditional tissue culture plates that do not accurately recapitulate the molecular and mechanical features necessary for modeling tissue- and organ-level functionalities. Overcoming these limitations requires the design and application of tunable biomaterial scaffolds. Silk fibroin is an attractive biomaterial due to its biocompatibility and versatility, which include its ability to form hydrogels, sponge-like scaffolds, and electrospun fibers and membranes appropriate for tissue engineering and biomedical applications. In this study, we show that hiPS cells can be differentiated into post-mitotic kidney glomerular podocytes on electrospun silk fibroin membranes functionalized with laminin. The resulting podocytes remain viable and express high levels of podocyte-specific markers consistent with the mature cellular phenotype. The resulting podocytes were propagated for at least two weeks, enabling secondary cell-based applications and analyses. This study demonstrates for the first time that electrospun silk fibroin membrane can serve as a supportive biocompatible platform for human podocyte differentiation and propagation. We anticipate that the results of this study will pave the way for the use of electrospun membranes and other biomimetic scaffolds for kidney tissue engineering, including the development of co-culture systems and organs-on-chips microphysiological devices.

## 1. Introduction

Podocytes are highly specialized epithelial cells that wrap around the glomerular capillaries and exhibit cellular protrusions known as foot processes [1]. Foot processes of neighboring podocytes form interdigitations and a slit diaphragm, which function together with the glomerular basement membrane and endothelial cells to form the glomerular filtration barrier. This multicomponent barrier plays a crucial role in retaining proteins and blood cells in the vasculature while removing toxins and metabolic waste products into the urinary filtrate [2]. Injury to podocytes results in impaired slit diaphragm and foot process effacement, which compromises the filtration function of the kidney and can lead to proteinuria, tissue degeneration, and kidney failure [3]. Studies aimed at understanding the onset and progression of kidney diseases have been conducted by using animal models and immortalized podocyte cell lines [4,5]. However, the current understanding of podocytopathies is limited by the lack of functional podocytes that can accurately model human biological responses and disease mechanisms. Recent advances in stem cell differentiation are helping to overcome this challenge by providing a highly efficient protocol to differentiate hiPS cells into functional human podocytes in tissue culture plates and organ-on-a-chip microfluidic devices [6,7,8]. These previously established protocols produce cells that exhibit primary and secondary foot processes and express podocyte markers, including Wilms Tumor 1 (WT1), nephrin, and podocin [7]. The establishment of a robust method to derive human mature podocytes from stem cells will enable more physiologically relevant studies of kidney function and disease pathogenesis.

Conventional tissue culture plates do not recapitulate the 3D structural features of tissues which can play key roles in mediating cell–cell interactions, nutrient transport, and cell signaling [9]. Thus, biomaterials provide alternative strategies for cell and tissue differentiation, as well as molecular signaling in a way that resembles in vivo cellular responses [10,11]. Biomaterials with unique microscale topographical features can facilitate podocyte adhesion and propagation. For example, Korolj and colleagues developed poly(dimethylsiloxane) (PDMS) substrates with convex surface topologies (~7.8 μm height and ~40.5 μm width) to mimic the surface topography of the glomerulus; podocytes cultured on these surfaces showed enhanced foot process interdigitation [12]. Three-dimensional alginate scaffolds with micro-convex topographies have also been used for podocyte culture and shown to enhance foot process interdigitations [13]. However, the podocytes used in these studies were derived from non-human sources, which limits the relevance and utility of these models for understanding human biological responses and disease. To overcome these limitations, synthetic (e.g., polylactic-co-glycolic acid [14,15] and polycaprolactone [16]) and naturally derived biomaterials (e.g., collagen [17]) have been widely investigated. While synthetic materials tend to be less biocompatible, naturally derived materials can suffer from limited mechanical properties, which lead to challenges in manufacturing, manipulation, and application for load-bearing purposes [18,19,20]. As a result, a material that possesses superior biocompatibility and mechanical properties would be highly attractive for kidney tissue engineering.

Silk fibroin (SF), a naturally derived protein polymer from Bombyx mori cocoons, has gained significant interest as biomaterial scaffolds due to its robust mechanical properties, biocompatibility, and biodegradability [21]. SF contains a light chain (~26 kDa) and a heavy chain (~390 kDa) linked by a disulfide bond, and the polymer is rich in hydrophobic β-sheet-forming domains that can self-assemble to form strong and resilient materials [22,23]. Additionally, SF is compatible with various processing techniques to generate numerous material formats, including hydrogels [24,25], thin films [26], nanoparticles [27], and electrospun membranes [28,29]. Among all these techniques, electrospinning has gained great attention for its ability to generate porous and fibrous nano-scale features that resemble the natural morphology of the extracellular matrix (ECM) in vivo [30]. Electrospun SF has been widely studied as tissue engineering scaffolds and in wound healing. For example, Tanzi and colleagues engineered electrospun SF vascular grafts with a small inner diameter (1.5 mm), and an in vitro study with primary porcine smooth muscle cells confirmed the cytocompatibility of the scaffolds [31]. Additionally, Correia and colleagues generated bi-layered electrospun SF membranes with polycaprolactone and hyaluronic acid in different layers, and the resulting membranes supported adhesion and spreading of human fibroblasts [32]. More recently, SF was coupled with decellularized ECM to generate electrospun matrices for islet survival [33]. Because most of these studies employed non-human cell sources or immortalized cell line [34,35], it remains unclear whether SF scaffolds can support the derivation and propagation of a wide variety of human cells, including human kidney podocytes. Additionally, studies utilizing hiPS cell differentiation techniques often obtain the final cell types by differentiating them in a tissue culture plate before seeding onto the SF substrates [36,37]. Thus, it is unclear whether electrospun SF can directly facilitate the differentiation and maturation of the human kidney podocytes in situ or enable the development of more sophisticated tissue culture systems, such as co-culture membranes and organs-on-chips microfluidic devices.

**Figure 1:**
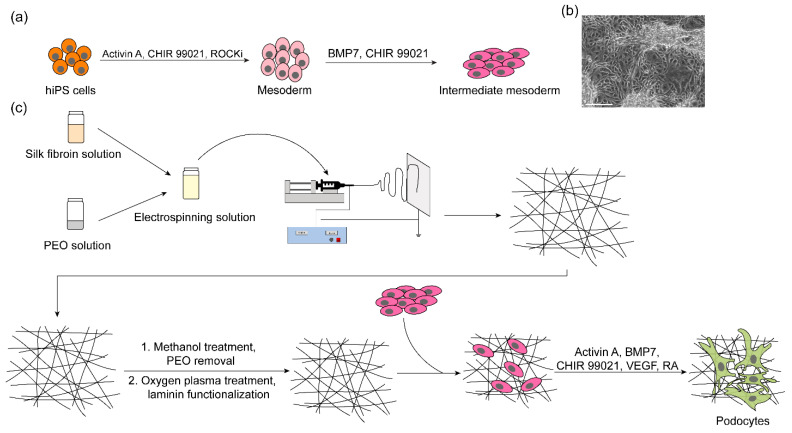
Schematic overview of the research study. (**a**) Differentiation of hiPS cells into intermediate mesoderm, the precursor cells from which kidney podocytes could be derived. (**b**) Representative bright field image of intermediate mesoderm cells derived from hiPS cells. Scale bar 550 µm. (**c**) Production of electrospun silk fibroin nanofibrous membrane and their use for cell adhesion and differentiation.

In this study, we generated electrospun SF membranes for the differentiation of mature human podocytes. The electrospun membranes exhibited nanofibrous morphology that resembles the porous features of the glomerular basement membrane. By following our previously established protocol for stem cell differentiation [6,7,8] (Figure 1a,b), we differentiated mature human podocytes on electrospun membranes functionalized with laminin-511 (Figure 1c). The differentiated podocytes developed foot process-like features and expressed podocyte-specific markers, including nephrin and podocin, as revealed by immunofluorescence microscopy, RT-qPCR, and Western blot analyses. Additionally, prolonged culture of the differentiated cells on the electrospun membranes in a maintenance medium demonstrated the ability of the scaffold to support long-term culture and viability of the cells while maintaining the expression of podocyte lineage identification markers. To the best of our knowledge, this is the first study demonstrating successful differentiation of mature hiPS cell-derived podocytes on a biomimetic electrospun matrix. Integration of the human podocyte differentiation technique and electrospun SF membranes provides unique opportunities and a new platform for studying podocyte development and function. Ultimately, we anticipate that the electrospun SF membranes could enable the fabrication of more advanced 3D systems for the development of physiologically relevant in vitro models of the human kidney glomerulus.

## 2. Materials and Methods

### 2.1. Preparation of Silk Fibroin Solution

A total of 5% (*w*/*v*) SF solution (Sigma, St. Louis, MO, USA) derived from Bombyx mori cocoons was dialyzed against 10% polyethylene glycol (PEG) (Sigma, St. Louis, MO, USA, MW 10,000) solution in 3 mL 3.5K MWCO Slide-A-LyzerTM dialysis cassettes (Sigma, St. Louis, MO, USA) for 20 h to prepare a concentrated SF solution. After dialysis, the resulting SF solution volume should be around 3.5 mL, which is approximately 14.29% in concentration. The concentrated SF solution was then re-diluted to 8% (*w*/*v*) with ultrapure water (Invitrogen, Waltham, MA, USA) and mixed with 10% (*w*/*v*) polyethylene oxide (PEO) (Sigma, St. Louis, MO, USA, MW~900,000) solution to obtain a final concentration of 6.37% (*w*/*v*) SF and 2% (*w*/*v*) PEO solution for electrospinning. PEO solution of 10% (*w*/*v*) was obtained by adding PEO to ultrapure water and stirring for 24 h at room temperature.

### 2.2. Electrospinning

The SF/PEO solution was loaded into a 10 mL syringe (BD Plastic, Franklin Lakes, NJ, USA) connected to a blunt 16-gauge stainless steel needle (Weller, Besigheim, Germany). The needle was connected to a high voltage power supply (Gamma High Voltage Research, Inc., Ormond Beach, FL, USA), and the solution was flowed at a constant rate of 0.015 mL/min using a syringe pump (Chemyx, Inc., Stafford, VA, USA). A grounded flat piece of non-stick aluminum foil (10 cm × 10 cm) was placed 15 cm from the needle and used as a collection plate for the electrospun fibers. A voltage of 11 kV was applied to the needle for 10 min.

The electrospun SFs were then attached to circular glass coverslips (18 mm diameter, Thermo Scientific, Waltham, MA, USA) such that the membrane covers the surface of the coverslip and the ends wrapped around the edges of the coverslip. The SF membranes were then plasma treated for 30 s at 100 W in an Emitech K-1050X plasma asher, followed by treatment with 90% methanol (Acros Organics, Waltham, MA, USA) for 20 min to induce β-sheet formation. After drying in a fume hood overnight, the coverslips were immersed in distilled water for 48 h at room temperature to remove PEO.

### 2.3. Human Induced Pluripotent Stem Cell Culture

The hiPS cell line PGP1 (the Personal Genome Project) [38] was propagated on tissue-culture-treated 6-well plates that were coated with Matrigel (BD Biosciences, Franklin Lakes, NJ, USA). The cells were cultured with mTeSR1 medium (Stem Cell Technologies, Vancouver, BC, Canada) and incubated at 37 °C with 5% CO_2_, and the cells were passaged every 4–5 days by treatment with StemPro accutase (Thermo Fisher Scientific, Waltham, MA, USA).

### 2.4. Differentiation of hiPS Cells into Intermediate Mesoderm (IM) Cells

Tissue-culture-treated plates (12-well polystyrene plates, VWR, Radnor, PA, USA) were incubated with 5 µg/mL laminin-511-E8 solution (iMatrix-511) in sterile water for 3 h at room temperature. hiPS cells were dissociated from Matrigel-coated plates by treatment with enzyme-free cell dissociation buffer (Thermo Fisher Scientific, Waltham, MA, USA) and centrifuged (Avanti J15-R, Beckman-Coulter, Pasadena, CA, USA) at 200 g for 5 min in DMEM/F12. Then, the cells were resuspended in a mesoderm differentiation medium containing DMEM/F12 with GlutaMax (GIBCO) supplemented with 100 ng/mL activin A (Thermo Fisher Scientific, Waltham, MA, USA), 3 µM CHIR99021 (Stemgent, Cambridge, MA, USA), 10 µM Y27632 (TOCRIS, Minneapolis, MN, USA), and 1 × B27 serum-free supplement (GIBCO, Waltham, MA, USA), and 100,000 cells were plated onto each well of a 12-well plate coated with the laminin-511-E8 solution. After 2 days of differentiation, the cell culture medium was switched to an intermediate mesoderm induction medium containing DMEM/F12 with GlutaMax supplemented with 100 ng/mL BMP7 (Thermo Fisher Scientific, Waltham, MA, USA), 3 µM CHIR99021, and 1 × B27 serum-free supplement, and the cells were incubated for a minimum of 14 days with daily medium replenishment.

### 2.5. Differentiation of IM Cells into Podocytes on Electrospun Silk Fibroin

The SF-covered coverslips (SFCCs) were treated with oxygen plasma for 60 s at 50 W in the Emitech K-1050X plasma asher to activate the SF surface with reactive hydroxyl groups. The SFCCs were subsequently rinsed with 70% ethanol once, followed by rinsing with sterile water three times. The resulting SFCCs were incubated with 25 µg/mL laminin-511 (Biolamina, Sundbyberg, Sweden) solution in 1×DPBS that contains Calcium and Magnesium (Gibco, Waltham, MA, USA) at 4 °C for 24 h. Freshly differentiated hiPS cell-derived IM cells were treated with 0.05% warm trypsin-EDTA for 5 min at 37 °C, individualized, followed by centrifugation at 200 g for 5 min in DMEM/F12. The cells were then re-suspended in a podocyte induction medium containing DMEM/F12 with GlutaMax supplemented with 100 ng/mL BMP7, 100 ng/mL activin A, 50 ng/mL VEGF (Thermo Fisher Scientific, Waltham, MA, USA), 3 µM CHIR99021, 1 × B27 serum-free supplement, and 0.1 µM all-trans retinoic acid (Stem Cell Technologies, Vancouver, BC, Canada). The IM cells were subsequently plated onto the laminin-coated SFCCs at a seeding density of 150,000 cells/mL. The cells were incubated at 37 °C with 5% CO_2_, and podocyte induction medium was changed every day for 5 days.

Differentiated podocytes were maintained in CultureBoost medium (Cell Systems, Kirkland, DC, USA) after the initial 5 days of podocyte induction at 37 °C with 5% CO_2_. The CultureBoost medium was changed every 2 days, and the cells were maintained in culture for an additional 14 days after the initial 5-day induction period.

### 2.6. Differentiation of IM Cells into Podocytes on Tissue Culture Plates

IM cells were seeded and differentiated into mature podocytes based on the protocol reported previously by Musah et al. [6,7]. The cells cultured on tissue culture plates were used as control and compared to the SFCC with regard to their ability to support the differentiation of podocytes. Twelve-well tissue culture-treated plastic plates (VWR, Radnor, PA, USA) were incubated with 5 µg/mL laminin-E8 (Takara, Denver, NC, USA) at 4 °C for 24 h or at room temperature for 3 h. The IM cells were seeded onto the laminin-coated plates at a seeding density of 150,000 cells/mL. The cells were incubated at 37 °C with 5% CO_2_, and podocyte induction media were changed every day for up to 5 days to induce differentiation of the IM cells into podocytes.

### 2.7. Attenuated Total Reflectance Accessory–Fourier Transformed Infrared (ATR-FTIR) Spectroscopy

Fourier transformed infrared spectroscopy (FTIR) analysis of the electrospun SF was performed using a Thermo Electron Nicolet 8700 (Thermo Fisher Scientific, Waltham, MA, USA) with an attenuated total reflectance accessory (ATR). Each spectrum was acquired in absorbance mode on a Ge crystal at a resolution of 4 cm^−1^ with 32 scans and a spectral range of 600 to 2000 cm^−1^. Background spectra were collected from the same circular glass coverslip on top of a non-stick aluminum foil prior to sampling. One sample was measured for each condition, and three different areas were measured on each sample. The average values of three areas were used for absorption peak analysis.

### 2.8. Profilometer

Square SF membranes (1 cm^2^) were placed on top of oxygen plasma-treated round coverslips (18 mm in diameter) without covering the entire coverslip surface. Prior to profilometer (Bruker Dektak 150, Bruker Inc., Billerica, MA, USA) characterization, the SF membranes were treated with 90% methanol and immersed in water as described above.

The SF membrane samples were loaded onto the equipment sample stage, and the stage location was adjusted to center the sample under the stylus. The sample thickness was measured by scanning the stylus alongside the coverslip surface to the SF membrane surface and back to the coverslip surface. The thickness readout was recorded by substracting the SF membrane height to the coverslip height. Four different measurements were performed on each sample, and two to three samples were used in each experiment. Two independent experiments were performed.

### 2.9. Scanning Electron Microscopy (SEM)

Electrospun SF membranes were imaged using scanning electron microscopy (SEM). To prepare for SEM imaging, the samples were mounted onto stubs using adhesive copper tape and sputter-coated with a thin layer of gold (12 mA, 300 s) in a Denton Desk V sputter unit (Denton Vacuum, Moorestown, NJ, USA). SEM images were then captured at 2 kV accelerating voltage and 25 pA emission current using an Apreo 2 scanning electron microscope (Thermo Fisher Scientific, Waltham, MA, USA) at the Duke Shared Materials Instrumentation Facility. The SF fiber diameters were measured from the SEM images using ImageJ (Version: 2.3.0/1.53f). A total of 500 randomly selected fibers were selected from five different SEM images to obtain fiber diameter distribution histogram of each sample. The fiber diameter histogram was analyzed and plotted using Microsoft Office Excel (version 2203) and Prism software (GraphPad Software, version 9.2.0.).

SEM was also employed to examine the morphology of differentiated podocytes cultured on electrospun SF. Firstly, human iPSC-derived podocytes were fixed in 2.5% glutaraldehyde in 0.1 M sodium cacodylate buffer (Electron Microscopy Sciences, Hatfield, MA, USA) for 1 h followed by rinsing with 0.1 M sodium cacodylate buffer (3×, 10–15 min each). Subsequently, the cells were post-fixed by 1% osmium tetroxide in 0.1 sodium cacodylate buffer (Electron Microscopy Sciences, Hatfield, MA, USA) for 1 h. After fixation, the samples were rinsed (3 times and 5 min each) with DI water, dehydrated in ascending grades of ethanol (30% for 5 min, 50% for 5 min, 70% for 5 min, 80% for 5 min, 90% for 10 min, 95% for 10 min, and 100% 10 min) and then chemically dried with hexamethydisilazane (Electron Microscopy Sciences, Hatfield, MA, USA) in a fume hood overnight. After sputter coating the samples with gold (12 mA, 300 s) in a Denton Desk V, the surface morphology of podocytes on electrospun SF was observed using the Apreo 2 scanning electron microscope (2 kV, 25 pA) at the Duke Shared Materials Instrumentation Facility.

### 2.10. Immunostaining and Microscopy Analysis

For immunostaining, cells were fixed with 4% formaldehyde and then permeabilized with 0.125% Triton X-100/PBS for 5 min, followed by blocking with 1% BSA/0.125% Triton X-100/PBS for 30 min. After rinsing (3 times at 5 min each) with 0.125% Triton X-100/PBS, the cells were incubated overnight at 4 °C with primary antibodies diluted in permeabilization buffer (1:200 dilution ratio). The primary antibodies used included nephrin (ARP, Waltham, MA, USA; GP-N2), podocin (Abcam, Boston, MA, USA; ab50339), and Pax2 (Invitrogen, Waltham, MA, USA; 71-6000). Following overnight incubation, samples were rinsed with permeabilization buffer (3 times for 5 min each) and then incubated with secondary antibodies conjugated to either Alexafluor-488 (Life Technologies, Waltham, MA, USA, A21202; at 1:1000 dilution ratio) or Alexafluor-594 (Life Technologies, A21203; at 1:1000 dilution ratio) for 1 h at room temperature. The cells were then counterstained with 4′,6-diamidino-2-phenylindole (DAPI, Invitrogen, Waltham, MA, USA, D1306) at 1:1000 dilution in water for 5 min. To quench SF autofluorescence, the samples were incubated in 0.3% *w*/*v* Sudan Black B (Sigma, St. Louis, MO, USA; 199664) (in 70% ethanol) solution for 3 min and then rinsed three times with PBS before imaging. Epi-fluorescent images were captured using an EVOS M7000 microscope (Thermo Fisher Scientific, Waltham, MA, USA) while confocal images were captured with a Zeiss 780 upright confocal system using a 20×/1.0 Water Zeiss W Plan-Apochromat 421452-9800 WD 1.8 mm lens (Zeiss, White Plains, NY, USA) at the Duke Light Microscopy Core Facility. Image processing and 3D reconstructions were performed using Fiji for ImageJ (Version: 2.3.0/1.53f).

### 2.11. Quantitative Real-Time PCR

RNA was extracted from the cells using the NucleoSpin^®^ RNA extraction kit from Macherey-Nagel (MN, Allentown, PA, USA) by following the protocol provided by the vendor. Briefly, podocytes differentiated on SFCC were harvested by incubation with pre-warmed 0.05% trypsin-EDTA solution for 3 min, followed by rinsing the SFCC with warm DMEM/F12. We found that the short 3 min Trypsin-EDTA incubation was effective for harvesting the cells from SFCCs without fragmentation of the electrospun fibers. The resulting cell suspension was centrifuged at 200× *g* for 5 min; the cell pellet was lysed by cell lysing buffer provided in the extraction kit; and the lysate was filtered, desalted, and treated with rDNase (provided in the extraction kit), followed by rinsing and eluting to obtain the resulting pure RNA solution. Podocytes differentiated on tissue culture plates (control substrate) were lysed directly in the plates after washing once with warm DMEM/F12. The RNA solution was quantified by NanoDrop One/OneC Microvolume UV-Vis Spectrophotometer (Thermo Fisher Scientific, Waltham, MA, USA). qRT-PCR was performed with the Luna Universal One-Step RT-qPCR kit (NEB, Ipswich, MA, USA) using the QuantStudio3 (Applied Biosystems, Waltham, MA, USA) with the following thermal cycling steps: 55 °C for 10 min, 95 °C for 1 min and 40 cycles of 95 °C for 10 s and 60 °C for 1 min according to manufacturer’s protocol. Primers used for the reactions are listed in Supplementary Table S1, and all target gene expression levels were normalized to GAPDH mRNA levels. All assays were performed in triplicates, and the data represent three independent experiments. qRT-PCR data were analyzed and plotted using GraphPad Prism software (Version 9.2.0).

### 2.12. Western Blot

Podocytes differentiated on SFCC were harvested following the same method that was used for RT-qPCR cell harvesting, and the cell pellet was lysed in RIPA supplemented with protease and phosphatase inhibitor cocktail (Roche, Indianapolis, IN, USA; 04 693 132 001 and 04 906 837 001) at 4 °C on a shaker for 30 min for protein extraction. Podocytes differentiated on tissue culture plates (control substrate) were also lysed by following the same procedure. Pierce BCA protein assay Kit (Thermo Fisher Scientific, Waltham, MA, USA) was used for protein quantification. A measurement of 7.5 ug protein lysate from each sample was separated onto a 4–15% stain-free gel (Bio-RAD, Hercules, CA, USA) by electrophoresis at 150 V for 50 min and transferred to 0.45 um PVDF membrane (BioRAD, Hercules, CA, USA). After transfer, the membranes were blocked with 5% Blutto/TBS-T for 30 min at room temperature and then incubated with primary antibodies in 5% Blutto/TBS-T at 4 °C overnight. The primary antibodies and their dilution ratios were anti-nephrin (ARP, Hercules, CA, USA; GPN02, 1:500), anti-podocin (abcam, Boston, MA, USA; ab50339, 1:500), anti-WT1 (Sigma, St. Louis, MO, USA; mab4234, 1:500), and anti-GAPDH (Invitrogen, Waltham, MA, USA; ma5-15738, 1:10,000). After overnight incubation, the PVDF membranes were washed (3 times, 10 min each) and then incubated with anti-guinea pig, anti-rabbit immunoglobulin G (IgG) horseradish peroxidase (HRP)-linked antibodies at room temperature for 1 h. All the membrane-washing steps were performed with Tris-buffered saline (BioRad, Hercules, CA, USA) supplemented with Tween 20 (Sigma, St. Louis, MO, USA) (TBST), and one single type of HRP-linked antibodies was used at a time. Immunoreactive bands were developed using SuperSignal West Femto substrate (Thermo Fisher Scientific, Waltham, MA, USA, 34095). The chemi-luminiscent signals were acquired using a GelDoc Imager (Bio-Rad, Hercules, CA, USA). All Blue Prestained Protein Standards (Bio-Rad, Hercules, CA, USA) were used for band referencing and blot orientation during electrophoresis and membrane transfer. The Western blot data were acquired from two independent experiments. Quantification of the band intensity was performed using ImageJ software (Version: 2.3.0/1.53f), and the band intensities were normalized to their corresponding GAPDH band intensities. Band intensities were plotted using GraphPad Prism software (Version 9.2.0).

### 2.13. CCK-8 Cell Viability Assay

Podocytes cultured on SFCCs and tissue culture plates were incubated for 2 h at 37 °C with 5% CO_2_ with CCK-8 (Sigma, St. Louis, MO, USA; 96992) reagent diluted in CultureBoost medium at a ratio of 1:10. A total of 100 µL of the mixture from each well was then transferred to a 96-well clear flat bottom plate (Greiner, Monroe, LA, USA; 655094) for absorbance measurement at 450 nm using a plate reader (FLUOstar OPTIMA, BMG LABTECH, Cary, NC, USA). CCK-8 data were collected from two samples of each condition, and two independent experiments were performed. For each time point, the relative podocyte viability was obtained by normalizing readings of podocyte on SFCCs to the readings of podocytes on tissue culture plates using Microsoft Office Excel (Version 2203), and the relative viabilities were plotted using GraphPad Prism software (version 9.2.0.).

### 2.14. Statistical Analysis

All data were presented as mean ± standard deviation unless stated otherwise. To compare two groups, the data were analyzed using two-tailed Student’s *t*-test analysis with a 95% confidence interval at assumption of equal variances. Differences were considered significant at * *p* < 0.05, ** *p* < 0.01, and *** *p* < 0.001. All statistical analysis was performed in GraphPad Prism software (version 9.2.0.).

## 3. Results and Discussions

### 3.1. Fabrication and Characterization of Electrospun Silk Fibroin Membranes

**Figure 2:**
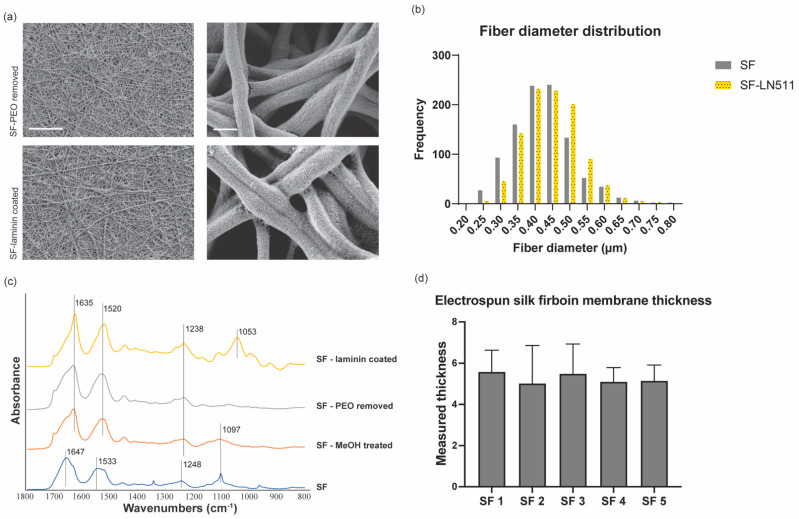
Molecular and physical characterization of the electrospun silk fibroin membranes. (**a**) Electron micrographs of the electrospun silk fibroin membranes. Top panel, silk fibroin membranes after removing PEO. Bottom panel, silk fibroin membranes after coating with laminin-511. Scale bars, 25 µm (left images), 500 nm (right images); the scale bars apply to all images in the same column. (**b**) Histogram of the electrospun silk fibroin nanofiber diameter distribution. Grey bars, silk fibroin after removing PEO. Yellow bars, silk fibroin after coating with laminin-511. (**c**) FTIR absorbance results of the electrospun silk fibroin membranes. The samples were characterized at multiple stages of the fabrication process, which includes directly after electrospinning (SF), after 20 min methanol treatment (SF—MeOH treated), after removing PEO (SF—PEO removed), and after coating with laminin-511 (SF—laminin coated). (**d**) Profilometer measurement of SF membrane thickness. SF 1 through 5 indicate average readings from five different membranes.

Uniform nonwoven nanofibrous SF membranes were generated by modulating electrospinning configurations, including voltage, extrusion speed, the distance between the needle tip and the collecting aluminum foil, and PEO concentration. PEO concentration played a key role in the generation of uniform nanofibers; at a low PEO concentration (5% *w*/*v*), we observed the formation of bead-like structures on the resulting nanofibers, as shown in Supplementary Figure S1a, where red arrowheads indicate examples of the bead defects on the electrospun nanofibers. By contrast, increasing the PEO concentration to 10% *w*/*v* enabled the successful generation of uniformly shaped nanofibers (Supplementary Figure S1b). To obtain nanofibrous membranes for cell culture, electrospinning was performed for 10 min to produce a dense (multi-layered) and porous SF substrate (Figure 2a) with an average fiber diameter of 0.41 ± 0.08 µm (Figure 2b).

The resulting SF membranes were treated with 90% methanol for 20 min to induce β-sheet conformation, which improves the mechanical properties of the electrospun SF membranes and prevents them from dissolving in aqueous environments [23] necessary for cell culture and tissue engineering applications. The β-sheet formation was confirmed by FTIR analysis, which revealed that the absorbance peaks of amide I (1647 cm^−1^), amide II (1533 cm^−1^), and amide III (1248 cm^−1^) shifted to the right of the spectrum, located near 1635 cm^−1^, 1520 cm^−1^, and 1238 cm^−1^, respectively (Figure 2c). PEO, which was added to the SF solution to facilitate electrospinning, was subsequently removed by immersion of the membranes in distilled water for 48 h. The removal of PEO was confirmed by the disappearance of the absorbance peak near 1097 cm^−1^, which is characteristic of C-O-C bond stretching vibrations (Figure 2c). Profilometer measurements revealed that the SF membranes had an average thickness of 5.27 µm (Figure 2d), which is significantly thinner than the widely used PDMS membranes (~50 µm) for tissue engineering in microphysiological systems. Thus, the engineered SF membrane could be potentially useful for modeling glomerular basement membrane, which is an ultrathin porous membrane that separates podocytes and glomerular endothelial cells in vivo [39].

In general, appropriate protein functionalization on biomaterial substrates is important for cell attachment, proliferation, and differentiation. For the differentiation of human podocytes, the electrospun SF membranes were functionalized with laminin-511 (25 µg/mL), a key component of the glomerular basement membrane. Successful coating of laminin-511 was confirmed by the appearance of an FTIR absorbance peak near 1053 cm^−1^, which is characteristic of C-N bond stretching vibrations (Figure 2c). Additionally, electron micrographs revealed a more textured morphology of the nanofibers coated with laminin, which presumably represents the presence of proteins adsorbed into the fibers (Figure 2a, bottom panel). The overall fiber density and diameter (0.40 ± 0.09 µm) were not significantly altered by functionalization with laminin (Figure 2a,b). These results show that electrospun SFs with multilayered nanofiber structures were successfully generated, and the membranes can be functionalized with basement membrane proteins for cell culture applications.

### 3.2. Differentiation and Characterization of Human Stem Cell-Derived Podocytes on Electrospun Membranes

**Figure 3:**
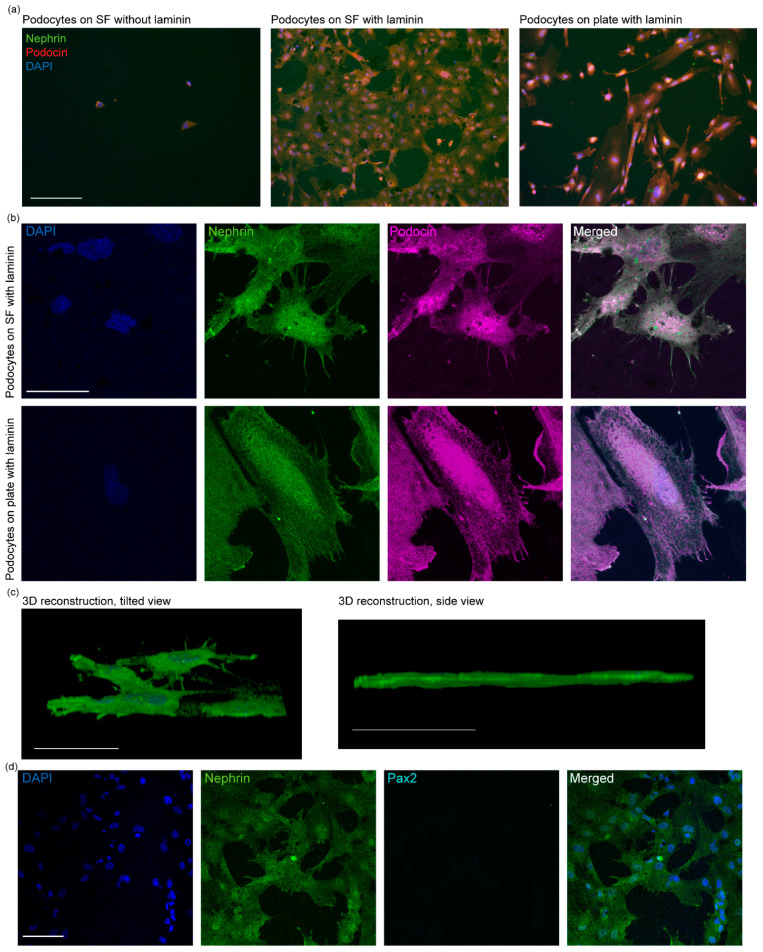
Immunofluorescence characterization of hiPS cell-derived podocytes differentiated on electrospun silk fibroin. (**a**) Florescence microscopy images of podocytes differentiated on electrospun silk fibroin membranes (left and middle) and plastic culture plates (right). Scale bar 275 µm. (**b**) Confocal images of podocytes differentiated on electrospun silk fibroin (top row) and on tissue culture plates (bottom row); scale bar 50 µm for all images. (**c**) Three-dimensional reconstruction of confocal microscopy images showing podocytes spreading and propagating on electrospun silk fibroin from tilted view (left) and side view (right); scale bar 50 µm. (**d**) Confocal microscopy images of podocytes differentiated on electrospun silk fibroin membranes showing loss of Pax2 as the cells specialize; scale bar 100 µm for all images.

We initially seeded hiPS cell-derived intermediate mesoderm cells onto the laminin-coated electrospun SF membranes and induced their differentiation to podocytes by treatment with a podocyte induction medium (containing Activin A, BMP7, CHIR 99021, VEGF, and retinoic acid) for 5 days. We also investigated whether SF membranes that were not functionalized with laminin could support the differentiation of podocytes, but the cells on these SF membranes were poorly attached and scarce, and exhibited small and rounded morphology, indicating that laminin coating of the SF membranes is essential for robust cell adhesion and podocyte differentiation (Figure 3a). As a positive control, we differentiated podocytes on tissue culture plates coated with laminin. Intriguingly, the electrospun SF membranes produced podocytes that were more robustly attached and spread out on the scaffold than those differentiated on traditional tissue culture plates (Figure 3a). Additionally, confocal microscopy images confirmed that the cells differentiated on the laminin-coated SF membrane expressed podocyte-specific markers, including nephrin and podocin (Figure 3b).

As podocytes mature during development, they form long cellular protrusions that extend from their cell bodies, known as foot processes [40]. These foot processes play important roles in podocyte physiology and kidney function. For example, the foot processes can enable podocyte interactions with the glomerular basement membrane and formation of interdigitated structure with neighboring podocytes to form the kidney’s blood filtration barrier [7]. Confocal microscopy analysis showed that the podocytes differentiated on the electrospun SF membranes developed foot processes, which also facilitated interaction between adjacent podocytes (Figure 3b). Three-dimensional reconstruction of the confocal microscopy images further revealed that the differentiated cells were well-spread and attached to the electrospun membranes, indicating that the electrospun SF membrane surface robustly supports podocyte differentiation and propagation (Figure 3c, left). We also observed that hiPS cell-derived podocytes remained on the apical side of the membrane, forming a monolayer of the cells without loss of cells through the pores of the scaffold (Figure 3c, right).

The development of foot processes in the podocytes differentiated on the SF membranes suggests that the cells have specialized features that would be necessary for modeling kidney function in more complex systems in vitro in the future. To further examine the maturity of the cells, we evaluated whether they expressed Pax2, which is an important transcription factor for kidney development [41] and widely expressed or transcriptionally active in developing or kidney progenitor cells but becomes significantly down-regulated when podocytes mature and acquire a functional phenotype. Thus, the loss or decrease in Pax2 expression is associated with podocyte maturation. We found that the hiPS cell-derived podocytes did not express Pax2 (Figure 3d), and Pax2 expression was observed only in the precursor cells (hiPS cell-derived intermediate mesoderm) (Supplementary Figure S2). These molecular characteristics are consistent with our previous observations for human podocyte differentiation in vitro as well as in intact human kidney glomerulus [7,42].

**Figure 4:**
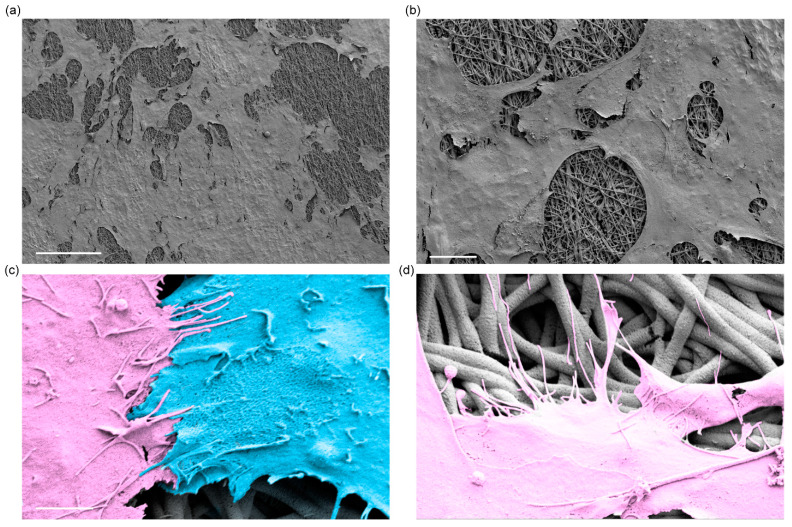
Scanning electron micrographs of hiPS cell-derived podocytes differentiated on electrospun silk fibroin membranes. (**a**) Representative view of podocytes adhered and spread on electrospun silk fibroin membranes; scale bar 150 µm. (**b**) Zoomed-in view of the podocytes on electrospun silk fibroin membranes; scale bar 25 µm. (**c**) Pseudo-colored electron micrographs showing interaction between adjacent podocytes via foot processes forming interdigitated-like structures; scale bar 2.5 µm. (**d**) Pseudo-colored electron micrographs showing foot processes of podocytes extending on and along the electrospun nanofibers; scale bar is the same as shown in image (**c**).

Furthermore, scanning electron microscopy images confirmed a high density of podocytes with arborized and well-spread cellular morphology on the electrospun membranes (Figure 4a,b). Examining the cells at high magnifications also confirmed that foot-like processes formed along and across the layers of electrospun fibers, and neighboring cells were interacting with each other through foot-like processes and formed an interdigitated-like structure (Figure 4c,d). These results suggest that podocytes were successfully differentiated and developmentally matured, generating foot-like processes to form an interdigitated structure with neighboring cells on the electrospun membranes. Thus, the differentiation of podocytes on the electrospun SF membrane produces cells that exhibit in vivo-like characteristics that could potentially guide the development of more complex and functional kidney models in the future.

### 3.3. Additional Characterization of Differentiated Human Podocytes through Gene and Protein Level Quantifications

**Figure 5:**
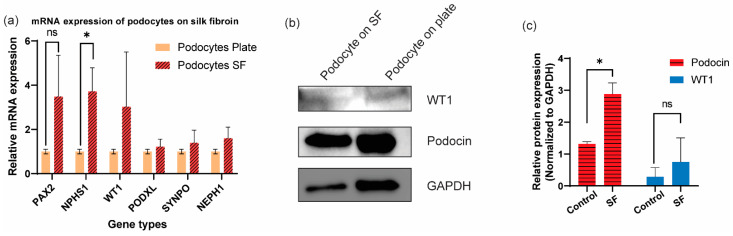
Quantification of podocyte-specific genes and proteins expressed in cells differentiated on electrospun silk fibroin. (**a**) RT-qPCR quantification of podocyte-specific genes. Yellow, podocytes differentiated on plastic culture plates; Red, podocytes differentiated on electrospun silk fibroin membrane. N = 3 independent experiments. ns, non-significant. * *p* < 0.05. (**b**) Western blot results of podocytes differentiated on electrospun silk fibroin membranes and tissue culture plates. (**c**) Quantification of Western blot bands with intensities normalized to GAPDH.

The cells differentiated on the electrospun SF expressed mRNA of important podocyte markers, including Wilms’ tumor 1 (WT1), podocalyxin (PODXL), synaptopodin (SYNPO), and NEPH1, at levels similar to those differentiated on laminin-coated tissue culture plates (positive control). Additionally, we observed similar levels of PAX2 mRNA expression between the cells on SF and tissue culture plate, indicating that the podocytes induced on SF are mature (Figure 5a) as previously reported for the control condition [6,7,8]. Intriguingly, the cells differentiated on SF expressed significantly higher levels of NPHS1 (nephrin) mRNA when compared to the cells differentiated on tissue culture plates (Figure 5a). Nephrin is an important podocyte-specific protein that mediates the formation of slit diaphragm between adjacent podocytes, which plays an important role in the regulation of the glomerular filtration barrier [43]. The higher mRNA expression of NPHS1 in podocytes that were differentiated on electrospun SF indicates that the SF substrate robustly supports the lineage specification and molecular characteristics of hiPS cell-derived podocytes and allowed the cells to become developmentally specialized.

Western blot analysis further confirmed the expression of two other important podocyte proteins, WT1 and podocin (Figure 5b). WT1 is essential for normal kidney development and has been found to play an important role in maintaining podocyte differentiation and function [44]. Additionally, WT1 is an upstream regulator of NPHS1 and PODXL [45], both of which are involved in the specification of podocyte phenotype. By quantifying the Western blot band intensities and normalizing them to GAPDH, we found comparable levels of WT1 expression between the cells differentiated on SF and tissue culture plates (Figure 5c). Podocin is another important podocyte-specific protein that localized to the cell body as well as the slit diaphragm. The expression of podocin at the protein level in the cells differentiated on SF further confirmed that the cells have specialized or are lineage restricted to the podocyte fate. Quantification of the Western blot data (Figure 5c) suggested that podocin expression in the cells on SF is significantly higher than the cells on tissue culture plates, which also supports the data presented thus far showing that the electrospun SF membrane facilitates the differentiation and maturation of hiPS cell-derived podocytes.

### 3.4. Long-Term Culture of Stem Cell-Derived Human Podocytes Differentiated on Electrospun Membranes

**Figure 6:**
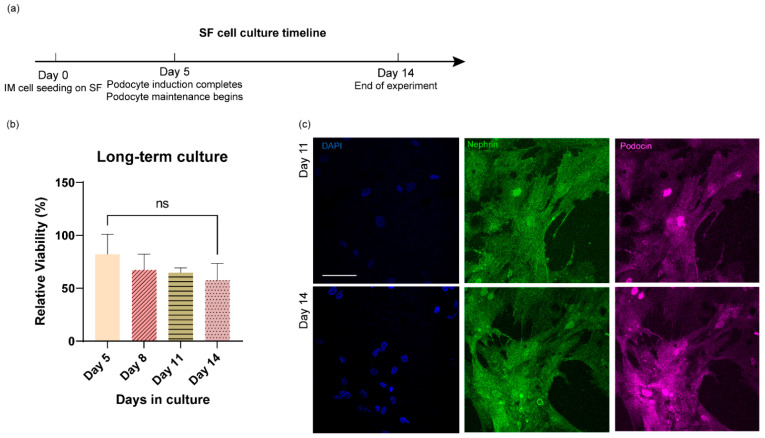
Long-term culture of hiPS cell-derived podocytes on electrospun silk fibroin. (**a**) Timeline for podocyte induction and prolonged culture on electrospun SF membranes. (**b**) CCK8 viability analysis of podocytes cultured on electrospun silk fibroin for 5, 8, 11, and 14 days. ns, not statistically significant. (**c**) Confocal images of podocytes cultured on electrospun silk fibroin for up to 11 and 14 days, scale bar 100 µm for all images.

We wondered if the hiPS cell-derived podocytes could be propagated long-term on the electrospun silk fibroin substrates after they had been fully differentiated into podocytes. To test this possibility, we differentiated the cells and cultured them for up to two weeks. We performed a cell viability assay at multiple time points, as well as immunofluorescence analysis. After 5 days of podocyte induction, the cells were maintained in CultureBoost medium, and the cell viability was measured every 3 days by a CCK-8 assay for up to 14 days (Figure 6a). The relative cell viability was calculated by comparing CCK-8 results obtained from podocytes differentiated on silk fibroin to the results obtained from podocytes differentiated on a tissue culture plate at each time point, and there was no statistically significant difference in the levels of cell viability during the longer-term cultures (Figure 6b). Additionally, confocal microscopy confirmed that the hiPS cell-derived podocytes maintained their morphology after the 14-day culture period (Figure 6c). On day 11 and day 14, the cell density remained high, the cells remained well-spread, and adjacent cells continued to interact with each other through their extended foot processes (Figure 6c). Together, these results indicate that the electrospun SF membranes support both the differentiation and long-term propagation of human iPS cell-derived podocytes, which could greatly advance kidney tissue engineering and related applications in the future.

## 4. Summary

In this study, we showed that laminin-coated electrospun SF supports the differentiation of hiPS cells into mature human kidney podocytes, as evidenced by the expression of cell lineage-specific markers, including nephrin and podocin, as well as the development of foot processes. The high expression of nephrin and podocin accompanied by the low expression of the progenitor cell marker Pax2 further validated the maturation of the hiPS cell-derived podocytes differentiated on the electrospun SF membranes. The SF membranes also facilitated long-term culture of the stem cell-derived human podocytes for at least 14 days while maintaining cell viability and adhesion. Previously, successful differentiation of mature podocytes was carried out on stiff and flat substrates such as traditional tissue culture plates or PDMS membranes embedded in microfluidic devices, both of which lack the biomimetic molecular and topographical features of natural basement membranes. The ability of electrospun SF to support podocyte differentiation, maturation, and long-term culture indicates that electrospun SF is a promising alternative biomaterial that could be useful as scaffolds for kidney tissue engineering and related applications in the future.

The limitations of the electrospun SF membrane used in this study include the opacity of the material, which can limit its applications when techniques or analyses require translucent material (e.g., live cell imaging). Thus, the SF membranes are better suited for end-point assays (e.g., immunostaining, Western blot, and gene expression analyses with either fixed or lysed cells). Additionally, the electrospinning process requires specialized equipment, such as high-voltage power supplies, that might not be readily available in many biology/bioengineering labs. We also acknowledge that the use of trypsin-EDTA to harvest podocytes from SFCC might lead to some loss of cell membrane proteins; thus, the optimization of reagent concentration and incubation time might be needed when interested in characterizing membrane proteins or harvesting cells for subsequent live-cell analysis. 

Future work could include integration of the electrospun SF membranes into microphysiological systems, such as microfluidic organs-on-chips devices, co-culture systems, and extracorporeal devices for modeling tissue structure, function, and disease phenotypes. The biomimetic SF membrane could also be used for modeling basement membrane dynamics in tissue development, physiology, and pathophysiology in vitro and in vivo.

## Data Availability

The data that support the findings of this study are available from the corresponding author upon a reasonable request.

## Supplementary Materials

The following supporting information can be downloaded at: https://www.mdpi.com/article/10.3390/bioengineering9050188/s1, Figure S1: Effect of silk fibroin electrospinning solution content on the resulting nanofiber morphology. (a) Electrospun silk fibroin with a low PEO content (5%), where bead-like defects can be observed. Red arrowheads, examples of beaded structure. (b) Electrospun silk fibroin with a high PEO content (10%), where uniform nanofibers were generated. Scale bar 2.5 µm; Figure S2: Immunostaining of hiPS cell-derived intermediate mesoderm cells for Pax2 (red, middle). Cells were counterstained with DAPI (blue, left). Merged view of the cells is shown on the right. Scale bar 275 µm; Table S1: List of the primers used in the RT-qPCR quantification experiment.
