# Supplementary material for: A Biomimetic Electrospun Membrane Supports the Differentiation and Maturation of Kidney Epithelium from Human Stem Cells
Source: Bioengineering (Basel). 2022 Apr 26;9(5):188. doi: 10.3390/bioengineering9050188 (PMC9137565; doi:10.3390/bioengineering9050188)
Supplement: Supplementary file 1 [file bioengineering-09-00188-s001.zip › bioengineering-1635315-supplementary.pdf]

Supplementary Information

A biomimetic electrospun membrane supports the differentiation and maturation of kidney epithelium from human stem cells

Authors

Xingrui Mou[1], Jessica Shah[1], Rohan Bhattacharya[1,2], Titilola D. Kalejaiye[1], Bowen Sun[3], Po-Chun Hsu[3], Samira Musah[1,2,4,5,6,*]

Author affiliations

[1]Department of Biomedical Engineering, Pratt School of Engineering, Duke University, Durham, NC 27708, USA

[2]Center for Biomolecular and Tissue Engineering, Duke University, Durham, NC 27708, USA

[3]Department of Mechanical Engineering and Material Sciences, Pratt School of Engineering, Duke University, Durham, NC 27708, USA

[4]Division of Nephrology, Department of Medicine, Duke University School of Medicine, Durham, NC 27710, USA

[5]Department of Cell Biology, Duke University, Durham, NC 27710, USA

[6]Affiliate Faculty of the Developmental and Stem Cell Biology Program, Duke Regeneration Center, and Duke MEDx Initiative
*Correspondence: samira.musah@duke.edu

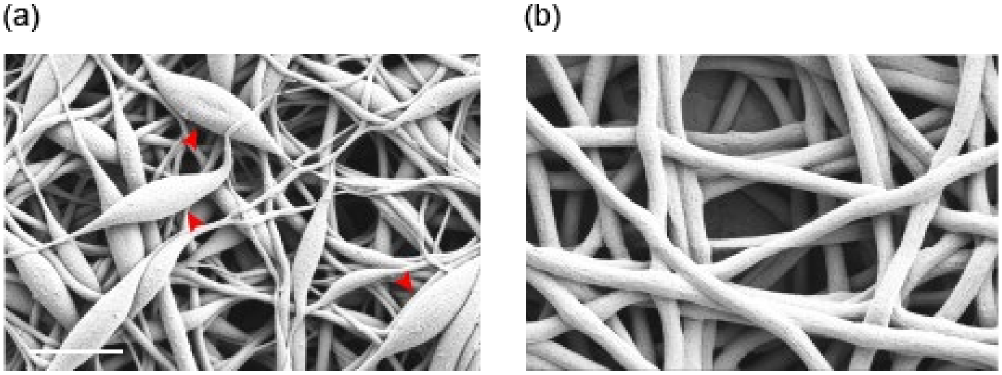

Figure S1. Effect of silk fibroin electrospinning solution content on the resulting nanofiber morphology. (a) Electrospun silk fibroin with a low PEO content (5%), where bead-like defects can be observed. Red arrowheads, examples of beaded structure. (b) Electrospun silk fibroin with a high PEO content (10%), where uniform nanofibers were generated. Scale bar 2.5 µm.

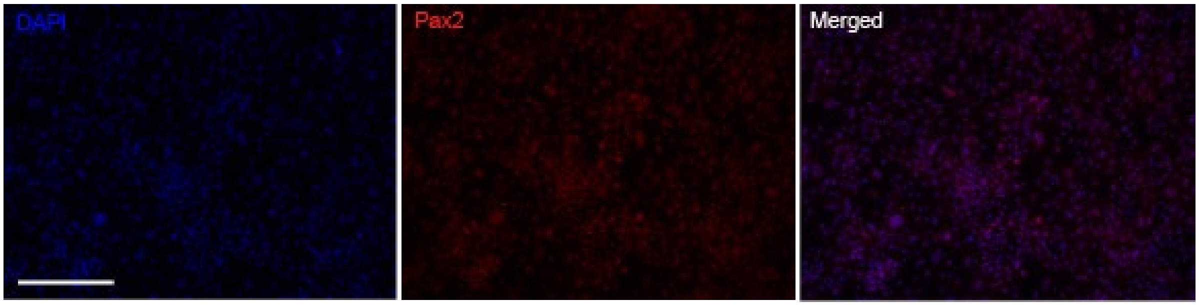

Figure S2. Immunostaining of hiPS cell-derived intermediate mesoderm cells for Pax2 (red, middle). Cells were counterstained with DAPI (blue, left). Merged view of the cells is shown on the right. Scale bar 275 µm.

Table S1. List of the primers used in the RT-qPCR quantification experiment.

Gene name	Name	Sequence
GAPDH	GAPDH-F	GGAGCGAGATCCCTCCAAAAT
	GAPDH-R	GGCTGTTGTCATACTTCTCATGG
PAX2	PAX2-F	AGATTCCCAGAGTGGTGTGG
	PAX2-R	CTCAAAGACCCGATCCAAAG
Nephrin	Nephrin-F	GCTTCTGCTCCTCTCCAATG
	Nephrin-R	CCCTGCCTCTGTCTTCTCTG
WT1	WT1-F	GGGTACGAGAGCGATAACCA
	WT1-R	TCTCACCAGTGTGCTTCCTG
Synaptopodin	Synpo S1	CATTCTGGAGGAGTCGATGG
	Synpo AS1	ATCCAGCAAGTCTGGATTCG
